# Challenges in investigating “Gut barrier dysfunction and bacterial translocation in ICU-acquired bacteremia”

**DOI:** 10.1186/s13613-024-01289-z

**Published:** 2024-05-26

**Authors:** Luping Wang, Xiaoxiao Xia, Qin Wu, Hao Yang

**Affiliations:** Department of Critical Care Medicine, West China Hospital, Sichuan University, Chengdu, China

## Dear editor

We read with great interest the recent article by Varkila et al. titled “Gut barrier dysfunction and the risk of intensive care unit (ICU)-acquired bacteremia - a case-control study” published in Annals of Intensive Care [1]. The authors investigated the relationship between gut barrier dysfunction and ICU-acquired bacteremia through a retrospective case-control study. While we appreciate their efforts to explore this important topic, several issues in the study design and interpretation of results warrant further discussion.

First, the authors used enterococcal bacteremia as a surrogate for bacterial translocation from the gut, with coagulase-negative staphylococcal (CoNS) bacteremia as a control representing non-gut origin infections. However, this assumption may be oversimplified. Enterococci can also contaminate catheters and cause bacteremia, while CoNS may occasionally colonize the gut. Completely attributing enterococcal bacteremia to gut translocation and CoNS bacteremia to catheter infection could bias the results.

Second, the biomarkers used in this study (intestinal fatty-acid binding protein (I-FABP), trefoil factor-3 (TFF3), and citrulline) may not accurately reflect gut barrier function. The authors observed unexpected positive correlations between citrulline and I-FABP/TFF3, suggesting these markers’ levels could be confounded by factors like renal dysfunction and critical illness severity [2]. Incorporating additional markers of gut permeability, mucus integrity, and mucosal immunity might provide a more comprehensive assessment of gut barrier dysfunction.

Third, the study only included ICU-acquired bacteremia occurring after 48 h of ICU stay, with a median enrollment time of 7 days post-ICU admission. This delayed timing may underestimate the risk of bacterial translocation during the acute phase of critical illness. Future studies should consider enrolling patients earlier and evaluating gut barrier function longitudinally.

Furthermore, blood cultures alone may not capture all bacterial translocation events, as the process can be transient and intermittent. Complementary approaches, such as detecting bacterial DNA in blood, analyzing the gut and blood microbiome, and assessing specific immune responses to translocated bacteria, could provide additional evidence of translocation [3].

Lastly, the retrospective case-control design has inherent limitations in controlling for confounding factors. Despite matching for age, renal dysfunction, and ICU length of stay, residual confounding might still exist. Stratified analyses, where the matching was broken, could be particularly prone to confounding. A prospective cohort study with comprehensive data collection and adjustment would be valuable to validate these findings.

In conclusion, while Varkila et al.‘s study provides valuable insights, further research is needed to elucidate the complex relationship between gut barrier dysfunction and ICU-acquired infections. A multi-faceted approach integrating clinical, microbiological, and immunological parameters may help to better characterize bacterial translocation and its clinical significance in critical illness.

## Data Availability

Not applicable.

## Abbreviations

ICU: Intensive care unit
CoNS: Coagulase-negative staphylococcal
I-FABP: Intestinal fatty-acid binding protein
TFF3: Trefoil factor-3
